# Plasma anandamide concentrations are lower in children with autism spectrum disorder

**DOI:** 10.1186/s13229-018-0203-y

**Published:** 2018-03-12

**Authors:** Debra S. Karhson, Karolina M. Krasinska, Jamie Ahloy Dallaire, Robin A. Libove, Jennifer M. Phillips, Allis S. Chien, Joseph P. Garner, Antonio Y. Hardan, Karen J. Parker

**Affiliations:** 1 Department of Psychiatry and Behavioral Sciences, Stanford University, 401 Quarry Rd., Stanford, CA 94305 USA; 2 Vincent Coates Foundation Mass Spectrometry Laboratory, Stanford University, 333 Campus Dr., Stanford, CA 94305 USA; 3 Department of Comparative Medicine, Stanford University, 287 Campus Dr., Stanford, CA 94305 USA

**Keywords:** Anandamide, Autism spectrum disorder, Blood biomarker, Cannabinoid

## Abstract

**Background:**

Autism spectrum disorder (ASD) is a neurodevelopmental disorder characterized by restricted, stereotyped behaviors and impairments in social communication. Although the underlying biological mechanisms of ASD remain poorly understood, recent preclinical research has implicated the endogenous cannabinoid (or endocannabinoid), anandamide, as a significant neuromodulator in rodent models of ASD. Despite this promising preclinical evidence, no clinical studies to date have tested whether endocannabinoids are dysregulated in individuals with ASD. Here, we addressed this critical gap in knowledge by optimizing liquid chromatography-tandem mass spectrometry methodology to quantitatively analyze anandamide concentrations in banked blood samples collected from a cohort of children with and without ASD (*N* = 112).

**Findings:**

Anandamide concentrations significantly differentiated ASD cases (*N* = 59) from controls (*N* = 53), such that children with lower anandamide concentrations were more likely to have ASD (*p* = 0.041). In keeping with this notion, anandamide concentrations were also significantly lower in ASD compared to control children (*p* = 0.034).

**Conclusions:**

These findings are the first empirical human data to translate preclinical rodent findings to confirm a link between plasma anandamide concentrations in children with ASD. Although preliminary, these data suggest that impaired anandamide signaling may be involved in the pathophysiology of ASD.

## Introduction

Autism spectrum disorder (ASD) is a neurodevelopmental disorder characterized by restricted, stereotyped behaviors and impairments in social communication [1]. ASD affects approximately 1% of US children [2], yet its underlying biological mechanisms are poorly understood. Identification of ASD biomarkers therefore is a public health priority, as this would enhance diagnostic accuracy and facilitate targeted therapeutic development. One biological system of increasing interest for ASD is the endogenous cannabinoid or endocannabinoid system [3, 4]. Endocannabinoids (eCBs) are a specialized class of lipid neuromodulators that regulate synaptic transmission, and play an important role in behavioral functions with relevance to ASD (i.e., cognitive function, emotional regulation, social functioning, motivation, and reward processing) [5].

Anandamide (AEA) is one of the most studied eCBs and has been implicated in several preclinical ASD models [6]. Specifically, mice with neurexin and neuroligin genetic mutations demonstrate disrupted tonic eCB signaling at the synapse. In humans, neuroligin gene mutations are associated with ASD risk, suggesting that eCB system dysregulation may also occur in ASD patients [7]. Moreover, four additional ASD rodent models which demonstrate ASD-related behavioral impairments (i.e., FMR1 knockout, BTBR strain, valproate acid exposed, and postnatal lipopolysaccharide administered) show improvements in social functioning and anxiety-like behavior following pharmacological AEA modulation [6, 8, 9]. These collective findings suggest that heterogeneity in ASD pathophysiology may have a point of convergence within the eCB system. Yet, despite the promise of these preclinical data, no studies to date have investigated AEA concentrations in humans with ASD.

One reason for this gap in knowledge is that the majority of preclinical AEA studies have used brain tissue (in part, due to AEA’s high stable abundance in this matrix). Opportunities to study human brain tissue are of course rare, yet alternative matrices which are more accessible (e.g., blood) present their own challenges. These include (1) selection and refinement of appropriate lipid extraction and purification methods for AEA and (2) the low circulating abundance of AEA in blood. The present study therefore was designed to address these barriers to scientific progress regarding the role of AEA in ASD. The aims of this project were twofold: (1) optimize lipid extraction and liquid chromatography-tandem mass spectrometry (LC-MS/MS) methodology to detect AEA (350 Da) concentrations in small volumes (~ 100 μl) of human blood and (2) quantify plasma AEA concentrations in children with ASD and neurotypical control children. We hypothesized that children with ASD would have lower plasma AEA concentrations compared to control children, in keeping with preclinical ASD findings.

## Methods

### Participants, recruitment, and eligibility criteria

Participants were *N* = 116 children (*N* = 60 children with ASD and *N* = 56 neurotypical control children), aged 3 to 12 years. Participants had been recruited as part of a previous study to investigate blood biomarkers and genetic variants in children with ASD [10]. Participants with ASD were primarily recruited through the Autism and Developmental Disorders Research Registry and by flyers posted in the Stanford University Autism and Developmental Disorders Clinic. Unrelated control participants were recruited through advertisements posted online or hardcopy in the surrounding community.

A comprehensive diagnostic evaluation was performed in children with ASD to confirm the accuracy of their existing diagnosis based on the *Diagnostic and Statistical Manual of Mental Disorders, Fourth Edition, Text Revision* (DSM-IV-TR) criteria [11]. This diagnosis was confirmed using research diagnostic methods (i.e., the Autism Diagnostic Instrument-Revised and the Autism Diagnostic Observation Schedule [12, 13]) by assessors trained by a research-reliable clinician. Study eligibility criteria were as follows. All participants were required to be (1) pre-pubertal, (2) in good medical health, (3) willing to provide a blood sample, and (4) capable of completing behavioral testing. Participants with ASD were included if they had a full-scale IQ of 50 and above. Control participants were included if they had an IQ score of 70 and above. Cognitive functioning was determined using the Stanford Binet 5th Edition [14]. ASD participant exclusion criteria included (1) a genetic, metabolic, or infectious etiology for ASD or (2) a DSM-IV-TR diagnosis of any severe mental disorder (e.g., schizophrenia, bipolar disorder). Participants using psychotropic medications were included if medications were stable for at least 1 week before blood collection. The most common medications in use by participants with ASD were stimulants (*n* = 10), selective serotonin reuptake inhibitors (*n* = 8), norepinephrine reuptake inhibitors (*n* = 5), and antipsychotics (*n* = 5). Controls were required to (1) be free of present or past neurological disorders; (2) be free of present or past psychiatric disorders on the basis of behavioral scales, a psychiatric evaluation, and, if needed, the Kiddie-Schedule for Affective Disorders and Schizophrenia for School-Aged Children [15]; (3) have no evidence of difficulty during gestation, labor, delivery, or immediate neonatal period, or abnormal neurological or developmental milestones; and (4) have no siblings with ASD.

### Sample preparation and quantification

Blood collection was performed between 10 am and 2 pm to control for any potential circadian rhythmicity in plasma AEA concentrations [16, 17]. Whole blood samples were collected into EDTA-treated vacutainer tubes and promptly centrifuged (1300×*g* at 4 °C for 10 min). The plasma fraction was aliquoted into polypropylene tubes and stored at − 80 °C until the morning of quantitative analysis. Plasma samples underwent lipid extraction with a modified salt-assisted liquid-liquid extraction (SALLE) [18]*.* During method optimization, comparison of traditional toluene liquid-liquid extraction to SALLE demonstrated that although both methods provided similar extraction yields (toluene vs SALLE, 85–90% vs 90–95%, respectively), SALLE was more reproducible, provided greater precision with decreased matrix effects, and had more efficient recovery. Commercially available stable isotope-labeled AEA-d8 (Cayman Chemicals; Ann Arbor, MI) was used as an internal standard (IS) for LC-MS/MS in creation of a calibration curve for quantitation of the analyte of interest, endogenous AEA. Plasma samples (100 μl/participant) were thawed in an ice bath (in less than 30 min), de-proteinized with 200 μl of acetonitrile containing 10 ng/ml internal standard solution mix and 50 μl of 5 M ammonium formate. Sample mixtures were then vortexed for 1 min before being spun at 13,000 rpm at 4 °C for 5 min. Organic layers from each sample were collected and transferred to autosampler vials in preparation for LC-MS/MS. Each sample was measured in triplicate. The samples could not be run as one group and, thus, were run in three sets.

The LC-MS/MS system was a TSQ Vantage triple quadrupole mass spectrometer coupled with an Accela 1250 HPLC system (Thermo Fisher Scientific, San Jose, CA). Baseline separation was achieved with gradient elution from an Acquity BEH C18 column (150 mm × 2.1 mm, 1.7 μm particle size) (Waters, Millford, MA). Calibration curve linearity was validated in spiked plasma from 0.25–10,000 pg/μl. The lower limit of AEA quantification was 50 fg on column. The total LC-MS/MS run was 8 min in duration, and samples were maintained at 4 °C throughout. Mobile phases were 0.1% formic acid (A) and acetonitrile containing 0.1% formic acid (B). Linear gradient conditions were as follows: 0–1 min, 50% B; 1–2 min ramp to 98% B; 2–4 min, 98% B; 4–5 min ramp back to 50% B; and 5–8 min equilibration at 50% B. The heated electrospray source was operated in positive ion mode. The triple quadrupole mass spectrometer was operated in selected reaction monitoring (SRM) mode for mass/charge transitions specific to AEA (*m/z*: 348.3 > 287.4, 203.4, 269.2, 91.0) and AEA-d8 (*m/z*: 356.25 > 294.3, 252.1, 206.1). Chromatograms were processed using Xcalibur software as well as visually inspected for inconsistencies.

### Calibration curves and linearity

Intrinsic to the consideration of AEA quantification is the “endogenous level challenge,” which relates to the difficulty encompassed by efforts to minimize contributions of sample matrix (e.g., plasma) effects through the use of a blank matrix for preparation of calibration standards in LC-MS/MS quantitation [19, 20]. It is known that for analytes of interest, such as AEA, that will be present at basal conditions, availability of a true blank matrix is rare. Thus, several strategies have been proposed to overcome this confound. The following strategy is a variation of the “authentic analyte in authentic matrix” approach and is related to the “standard addition” approach [19, 20]. For the calibration curve, peak area ratio of spiked unlabeled to labeled analyte (AEA/D8-AEA) was determined in buffer (i.e., phosphate-buffered saline, termed PBS) and extracted “blank pooled plasma” (i.e., from non-affected, healthy adult plasma) and used to construct a linear regression equation: *y* = *m* (*x*) + *b*, where *y* is equal to the peak area ratio of spiked analyte/internal standard, *m* is equal to the slope of the calibration curve, *x* is equal to the concentration of analyte, and *b* is equal to the *y*-intercept of the calibration. Calibration curves were fitted using different weighting schemes (unweighted, 1/*x*, or 1/*x*^2^) and over different ranges of spiked AEA concentrations (minimum 0.1 pg/μl; maximum between 20 and 10,000 pg/μl prepared in plasma, equivalent to 0.5 pg to 50 ng on column). As experimental samples produced calculated AEA concentrations near the lower end of this range, the curve that generated the lowest average relative error at the lower end of the spiked concentration range was retained (range 0.2–20 pg/μl, weighted by 1/*x*^2^). Unknown sample AEA concentrations were corrected for the endogenous presence of the analyte in plasma, estimated as the negative *X*-intercept of the calibration curve. The lowest limit of quantification (LLOQ) in extracted plasma was defined as signal-to-noise ratio of 10 to 1 and was 50 fg for AEA. Additionally, the triplicate measures for each participant were highly consistent, with intra-class correlation (ICC) coefficients within each sample set between 90.2 and 94.8%. High ICC coefficients provide evidentiary support for the reliability of calculated AEA concentrations [21].

### Statistical analysis

Study data were managed using REDCap [22] and analyzed using JMP V.13 (SAS Institute, Cary, NC, USA). Four participants were excluded from analyses because their samples either produced highly inconsistent concentrations between replicates (*N* = 1F with ASD) or extremely elevated AEA outlier concentrations were detected (i.e., three standard deviations above mean; *N* = 3 controls: 2M, 1F). A logistic regression was performed to test whether mean AEA concentrations predicted group membership (i.e., ASD vs. control), using a generalized linear model with a binomial error distribution and logit link function. All models included the following blocking factors: age, full-scale IQ, ethnicity, gender, sample set, and sample order within set (as described below).

## Results

Characteristics of participants included in the statistical analyses are provided in Table 1. Full-scale IQ and age differed between children with and without ASD. To eliminate the possibility that these (or any other variables) could exert confounding effects and generate false positive or false negative results [23, 24], we adopted the standard epidemiological approach to this problem and included these variables in the statistical models as blocking factors. Additionally, secondary analyses were performed to determine whether blocking factors were related to plasma AEA concentrations. Plasma AEA concentrations were not predicted by age (*F*_1,107_ = 1.54, *p* = 0.218) or sex (*F*_1,107_ = 1.36, *p* = 0.246) alone. We also verified that blood AEA concentrations were not significantly related to blood collection time (*F*_1,98_ = 0.5595; *p* = 0.4563), including when analysis was performed using a quadratic term (*F*_1,98_ = 0.0417 *p* = 0.386).

**Table 1:** Participant characteristics

Demographics	Control children	Children with ASD
*N*	53	59
Female	20	14
Male	33	45
Age	7.13 ± 2.96	8.25 ± 2.67*
Full-scale IQ	115.49 ± 9.51	83.24 ± 28.37*
Full-scale IQ range	92–134	50–140
*N* cases with IQ < 70	0	17
*N* cases of psychotropic medication use	0	29
Ethnicity		
Asian	6	11
Caucasian	38	35
Other	9	13

Values in the table are reported as arithmetic means ± standard deviation. The *χ*^2^ likelihood ratio was used to examine whether the distribution of individuals in the two groups differed by sex and by ethnicity; no significant effects were found (sex, *χ*^2^(1) = 2.60, *p* = 0.107; ethnicity, *χ*^2^(2) = 2.03, *p* = 0.363). Welch’s unequal variances *t* test was used to test for differences in age and full-scale IQ between groups (* = *p* < 0.05). Significant group differences were observed for both measures (age, *t*(105.4) = 2.09, *p* = 0.039; IQ, *t*(72.2) = 8.23, *p* < 0.0001) and therefore were used as blocking variables in the analysis

*Abbreviations*: *ASD* autism spectrum disorder, *IQ* intelligence quotient

**Fig. 1:**
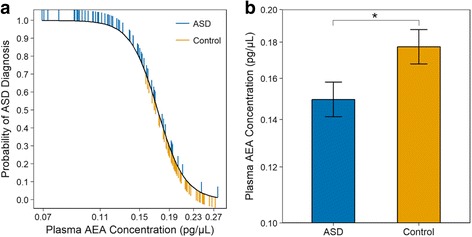
Plasma AEA concentrations in children with ASD and neurotypical control children. **a** Plasma AEA concentration significantly differentiates cases from controls. Plasma AEA is plotted partialed (adjusted) for other variables in the statistical model. ASD individuals plotted above, and control individuals plotted below, the dashed lines are correctly classified. **b** Plasma AEA concentrations in children with and without ASD, back-transformed from logged values. Data are presented as LSM ± SEM. Children with ASD had lower plasma AEA concentrations (mean 0.149 pg/μl) compared to control children (mean 0.177 pg/μl)

Plasma AEA concentrations significantly differentiated cases and controls (likelihood ratio chi-square, *χ*^2^ = 4.16, *p* = 0.041; Fig. 1a). Across the range of observed plasma AEA concentrations, the likelihood of ASD decreased over 20-fold, corresponding to nearly a fourfold decrease in risk with each twofold increase in plasma AEA concentration (range odds ratio = 0.043; unit odds ratio = 0.257; regression coefficient ± SE = − 1.359 ± 0.698). We next tested whether children with and without ASD differed in AEA concentrations. As predicted, plasma AEA concentration was significantly lower in ASD compared to control children (*F*_1,102_ = 4.64, *p* = 0.034; Fig. 1b).

Most experimental samples had calculated AEA concentrations that were higher than the LLOQ, but lower than the endogenous concentration for the AEA-spiked plasma matrix used in calibration. Therefore, to provide secondary support of the calculated AEA concentrations in patient samples, all analyses were also performed using ranks of AEA concentrations (instead of mean log-transformed concentrations) to verify that the results were robust deviations from linearity. Analyses using AEA concentration rank instead of AEA concentrations themselves produced qualitatively identical results and support the reliability of quantified AEA concentrations.

## Discussion

There is a growing body of preclinical data that suggests the eCB system may be involved in ASD pathophysiology. Specifically, AEA signaling has been shown to exert a modulatory role in rodent behaviors that are relevant to ASD symptomatology and to pharmacologically rescue the social deficits observed in ASD rodent models [25, 26]. The present study is the first to translate these preclinical data to patients, by optimizing a LC-MS/MS method to quantitatively analyze AEA concentrations in small volumes of banked plasma with short sample preparation time and high sample repeatability. Two significant findings were observed: (1) plasma AEA concentrations significantly differentiated ASD cases from controls, such that children with lower AEA concentrations were more likely to have ASD, and (2) AEA concentrations were significantly lower in ASD compared to control children (Fig. 1). These results, although preliminary, corroborate preclinical evidence that AEA signaling may be impaired in patients with ASD.

Detection of biomarkers in plasma is highly advantageous for brain disorders due to the relatively non-invasive procedures required to collect blood (compared with those required to access brain-relevant tissues). Circulating eCB concentrations are thought to be in equilibrium with brain-related eCB concentrations [27], suggesting that plasma eCB concentrations may be a viable proxy for the behavioral effects generated by bioactive, brain-related AEA. Therefore, although plasma AEA concentrations do not perfectly classify idiopathic ASD and control participants, AEA signaling impairments nevertheless may characterize a specific subset of ASD patients or be useful for inclusion in a multidimensional biomarker panel employed to detect idiopathic ASD.

The present findings are preliminary and warrant subsequent replication in an independent study cohort. These findings must also be considered in the context of several limitations. First, our study was not powered to assess potential sex differences and relationships with between behavioral symptomology and AEA concentrations within the heterogeneous ASD population. Second, our ASD participants were taking a variety of prescription medications in contrast to the control participants, which were medication free (Table 2). Thus, it is possible that our findings were driven by medication status, particularly as inclusion criteria only required medication to be stable for 1 week prior to blood collection. We think this is unlikely in light of the aforementioned preclinical AEA data (in which “affected” mice were medication-free); nevertheless, future research is required to evaluate the impact of commonly prescribed ASD medications on plasma AEA concentrations and other eCBs. Finally, we measured a single eCB analyte using extrapolated AEA concentrations. Further optimization of the present LC-MS/MS methodology would allow for more precise AEA quantitation as well as concomitant study of additional eCBs, such as 2-arachidonoylglycerol (2-AG), which has also been implicated in preclinical ASD models with respect to pathophysiology and behavioral features. AEA concentrations have been studied in other brain disorders and have been reported to be lower in patients with temporal lobe epilepsy and post-traumatic stress disorder [28, 29]. Whether the lower plasma AEA concentrations observed in ASD patients in the present study are related to common, associated features (e.g., epilepsy, anxiety), or core symptoms, remains to be determined.

**Table 2:** Participant medications

Medication class	Medications prescribed	Percent use in ASD (*n*)
α2A agonist	Guanfacine, Tenex, Clonidine	6.78% (4)
Antipsychotic	Abilify, Risperdal, Seroquel	8.47% (5)
Benzodiazepine	Lorazepam	1.69% (1)
NEI	Atomoxetine	8.47% (5)
SSRI	Citalopram, Escitalopram, Paroxetine, Sertraline, Fluoxetine	13.56% (8)
Stimulant	Mehtylphenidate, Dexmethylphenidate, Amphetamine salts, Lisdexamfetamine	16.9% (10)

Approximately 55.9% of the participants with ASD analyzed were using psychotropic medications at the time of blood collection

*Abbreviations*: *α2A agonist*, alpha-2 adrenergic agonist, *NEI* norepinephrine reuptake inhibitor, *SSRI* selective serotonin reuptake inhibitory

## Conclusion

In conclusion, this report extends preclinical findings to provide the first empirical evidence that plasma AEA concentrations are lower in individuals with ASD compared to neurotypical control individuals. Future research must now determine the relationship between plasma AEA concentrations and ASD symptom severity, particularly with regard to the core and associated features thought to be related to AEA signaling deficits in patients with ASD (i.e., phenotypic profiles with atypical cognitive and social functioning as measured by gold-standard assessments like the Autism Diagnosis Observational Schedule). In parallel, research is also needed to identify the mechanisms responsible for the lower AEA concentrations observed in ASD patients (e.g., is this reduction related to changes in AEA transporter proteins, synthesizing enzymes, catabolizing enzymes, and/or eCB receptor expression? [8, 25, 26, 30–32]). Should these follow-up studies implicate a convincing role for AEA in the pathophysiology of ASD, the eCB system may represent a promising target for therapeutic development in ASD.

## Abbreviations

2-AG: 2-Arachidonoylglycerol
AEA: Anandamide
ASD: Autism spectrum disorder
DSM-IV-TR: Diagnostic and Statistical Manual of Mental Disorders, Fourth Edition, Text Revision
eCB: Endocannabinoid
EDTA: Ethylenediaminetetraacetic acid
F: Female
FMR1: Fragile X Mental Retardation knockout mice
ICC: Intra-class correlation
IS: Internal standard
LC-MS/MS: Liquid chromatography with tandem mass spectrometry
LLOQ: Lowest limit of quantification
M: Male
PBS: Phosphate-buffered saline
SALLE: Salt-assisted liquid-liquid extraction
SRM: Selected reaction monitoring
